# Towards a needs-based design of the physical rehabilitation workforce in South Africa: trend analysis [1990–2017] and a 5-year forecasting for the most impactful health conditions based on global burden of disease estimates

**DOI:** 10.1186/s12889-021-10962-y

**Published:** 2021-05-13

**Authors:** Q. Louw, K. Grimmer, K. Berner, T. Conradie, D. T. Bedada, T. S. Jesus

**Affiliations:** 1 Division of Physiotherapy, Department of Health and Rehabilitation Sciences, Faculty of Medicine and Health Sciences, Stellenbosch University, PO Box 241, Cape Town, 8000 South Africa; 2 Division of Epidemiology and Biostatistics, Department of Global Health, Faculty of Medicine and Health Sciences, Stellenbosch University, Cape Town, South Africa; 3 Global Health and Tropical Medicine (GHTM) & WHO Collaborating Centre for Health Workforce Policy and Planning, Institute of Hygiene and Tropical Medicine - NOVA University of Lisbon (IHMT-UNL), Rua da Junqueira 100, 1349-008 Lisbon, Portugal

**Keywords:** Global burden of disease, Rehabilitation workforce, Human resources, YLDs

## Abstract

**Background:**

Rehabilitation can improve function in many people with chronic health conditions. It is important to consider priority conditions requiring rehabilitation, so it can be realistically positioned and costed in national health financing systems like South Africa (SA)‘s proposed National Health Insurance (NHI). This paper describes temporal trends of top-ranked conditions on years lived with disability (YLDs) rates in SA, for which physical rehabilitation can ameliorate associated disability.

**Methods:**

This study is a systematic synthesis of publicly available Global Burden of Disease (GBD) 2017 estimates. The top 11 conditions contributing most to YLDs and for which evidence-based rehabilitation interventions exist were identified. Age-standardized rates per 100,000 and YLDs counts were extracted from 1990 to 2017. Significance of changes in temporal trends was determined using Mann-Kendall trend tests. Best-fit rates of yearly changes were calculated per condition, using GBD estimates (2012–2017), and extrapolated (by imposing the best-fit regression line onto results for each subsequent predicted year) as forecasts (2018–2022).

**Results:**

Trends for YLDs counts per condition year (1990–2017) and forecasted values (2018–2022) showed an overall steady increase for all conditions, except HIV and respiratory conditions. YLDs counts almost doubled from 1990 to 2017, with a 17% predicted increase from 2017 to 2022. The proportionate contribution to YLDs counts reduced over time for all conditions, except HIV. Although age-standardized YLDs rates appear relatively stable over the analyzed periods for all conditions (except HIV, respiratory conditions and type 2 diabetes), trend changes in YLDs rates over 28 years were significant for all conditions, except neonatal (*p* = 0.855), hearing loss (*p* = 0.100) and musculoskeletal conditions (*p* = 0.300). Significant trend decreases were apparent for 4/9 conditions, implying that another 5/9 conditions showed trend increases over 28 years. Predicted all-age prevalence in 2022 suggests relatively large increases for cardiovascular disease and heart failure, and burns, while relative decreases are predicted for fractures and dislocations, stroke, and musculoskeletal conditions.

**Conclusion:**

Rehabilitation needs in SA are potentially massive and unmet, highlighting the need for innovative and context-specific rehabilitation that considers current local needs and projected changes. These findings should be considered when designing the NHI and other schemes in SA to ensure human and financial resources are deployed efficiently.

**Supplementary Information:**

The online version contains supplementary material available at 10.1186/s12889-021-10962-y.

## Introduction

South Africa is classified as an upper-middle-income country. This obscures its significant wealth inequality, where approximately 80% of people live in poverty with poor social circumstances such as high crime and violence levels [1–4]. Concomitant low education levels make it difficult to break out of the poverty cycle, limiting upskilling and employment opportunities [2]. This reflects in the South African 2019 unemployment rate (29.1%; before the Coronavirus disease [COVID-19] outbreak), one of the highest globally [5].

The private healthcare sector serves 20% of the South African population, but employs 70% of the country’s doctors and attracts over 50% of its healthcare funding. The remaining 80% of the population access the poorly-resourced public healthcare sector [6]. Over the past 3 years, South Africa’s economic growth has slowed to less than 1% and public debt levels are growing across all sectors. Many people are on waiting lists for essential services and the situation is worsening due to COVID-19. Predicted budget cuts over the next few years will further strain the health sector.

South Africa’s complex and unique health challenges are characterized by poor social determinants of health and a quadruple burden of disease (BoD). Paradoxically, the country’s health challenges have been further complicated by improved outcomes of pharmaceutical management for infectious diseases (HIV/AIDS and tuberculosis [TB]) as more people are now living with such chronic health conditions that were previously fatal. Whilst people with chronic infectious diseases may be living longer, they often suffer from compromised physical and mental functioning, disabilities and poorer quality of life, as a result of underlying disease processes and the ramifications of medication regimes [7]. There are also increasing reports of unanticipated comorbidities and disease interactions. For example, people living with chronic HIV are more susceptible to suffering stroke, whilst people living with chronic TB are more susceptible to deafness, chronic lung disease, or musculoskeletal pain [8–13].

The disease cascade and BoD consequently impact significantly on people’s wellbeing and social participation [14, 15]. Many are forced to rely on social grants because they are unable to work, and some require daily assistance from their families for basic activities of living, which limits the earning potential of caregivers [16].

Rehabilitation is the key health strategy to address compromised functioning and quality of life [17]. Recently, the World Health Organization (WHO) launched an international campaign (Rehabilitation 2030 [17]) that recognizes the importance of rehabilitation to optimize functioning. Population rehabilitation needs are increasing rapidly in low- and middle-income countries [18–20]. The WHO rehabilitation strategy emphasizes that accessible, safe and affordable rehabilitation is vital in achieving the third sustainable development goal: “ensuring healthy lives and promote well-being for all, at all ages” [21]. These aims concur with South Africa’s Constitution and the country’s White Paper on Disability Rights, which state that “all people with disability have the right to quality and accessible healthcare and rehabilitation” [22]. To achieve global and national goals, the WHO envisions that rehabilitation should be integrated across the continuum of care and should be funded by national health insurance systems. How this will play out in South Africa is unclear, given the limited allocation in health or social services budgets to support rehabilitation [23]. The situation may worsen when unemployment, compromised social circumstances, and mental health issues arising from COVID-19 are fully realized.

South Africa’s first National Health Insurance (NHI) Bill (11 of 2019) was published in August 2019 [24]. The NHI scheme aims to provide universal access to essential healthcare, regardless of needs, employment status or ability to contribute. Universal access to rehabilitation in South Africa should be considered carefully, so it can be realistically positioned, costed, and included in the proposed NHI scheme, in a way that matches key South African health and rehabilitation needs. Prioritization may need to occur due to scant funds for rehabilitation. Globally, the WHO has prioritized key conditions for which rehabilitation should be provided [25]. However, South Africa’s unique epidemiological profile requires specific consideration of rehabilitation needs. A recent cross-national comparison of physical rehabilitation needs across the BRICS nations (Brazil, Russian Federation, India, China, and South Africa), using Global Burden of Disease (GBD) estimates up to 2017 [18], found substantial differences in the evolution of rehabilitation needs. For example, a quarter of South African physical rehabilitation needs came from HIV-related conditions, while this approximated 1% for all other BRICS nations [18]. This analysis highlighted the importance of better understanding South Africa’s unique epidemiological and socio-economic contexts, to assist in understanding the coverage of current rehabilitation services [13, 26–30].

In this paper, we specifically assessed temporal trends (1990–2017) of GBD estimates of the most common conditions (as ranked by years lived with disability [YLDs] for South Africa) that could be improved by rehabilitation. We further forecast rehabilitation needs for 5 years (2018–2022), based on recent trends (2012–2017). The current trend analysis extends from previously published comparative analyses across the BRICS nations [18], to provide a more detailed synthesis with a focus on conditions unique to the South African context that contribute most to YLDs in this specific setting. As our synthesis incorporates the top conditions (based on their ranking by YLDs for South Africa specifically) for which evidence-based rehabilitation interventions exist to address associated disability, this paper reports on a different set of conditions (versus the BRICS paper), that are relevant to South Africa. While the paper on the BRICS nations aimed to compare regions, this paper focuses on understanding current rehabilitation service implications for the South African context based on a trend analysis and short-term 5-year forecasting extrapolated from recent historical trends (which was also not incorporated in the BRICS paper). Since South Africa is on the brink of moving towards NHI, this paper is important for designing local rehabilitation services.

## Methodology

### Design

A systematic synthesis of secondary, country-specific (for South Africa), publicly available GBD 2017 estimates was done [31].

#### Building the dataset

The GBD Study, initiated in 1990 and the most comprehensive epidemiological study to date, analyzes input sources to estimate mortality, causes of death and illness, and risk factors. The detailed methodology is described in [32]. This paper reports on freely available GBD estimates (http://ghdx.healthdata.org/gbd-results-tool); specifically, prevalence and YLDs. YLDs are estimates of the burden of non-fatal disease and injury and are calculated by multiplying the prevalence of each sequela by the estimated level of health loss in the form of a disability weight (DW). DWs range from 0 (i.e., perfect health) to 1 (i.e., death) to represent the severity of the disease, and were derived from population surveys using pairwise comparison methods between random pairs of health states [31, 33]. YLDs is a widely used, aggregative metric to quantify the disability burden; accounting for the prevalence of a condition, the resultant sequelae, the time the person lives with the sequelae, and the severity of those sequelae reflected into an assigned DW. Details on how YLDs and DWs are determined, along with the DWs for all conditions, are available elsewhere [32–34].

We first determined which conditions were highest-ranked based on YLDs rates in South Africa according to GBD 2017 estimates, which were the latest available at the time of the analyses. We initiated the process with a list of conditions used in previous physical rehabilitation needs analyses across nations [20], and for which evidence of physical rehabilitation effectiveness was found [20]. To that list, hearing loss and type 2 diabetes (DM2) were added. Hearing loss was added considering that it is a common multidrug-resistant TB medication side-effect [10]. Rehabilitation therapists are often involved in hearing screening and referral [35, 36]. DM2 was added as rehabilitation is an important component of its chronic management in South Africa [37, 38] and rehabilitation specialists are routinely involved in DM2 care at primary care level [39]. In accordance with its national policy to integrate chronic care at primary care level, the National Department of Health has established multidisciplinary chronic disease management teams that include rehabilitation specialists, and that are primarily responsible for DM2 care at primary care clinics [39]. DM2 is also prevalent in various rehabilitation contexts (e.g. cardiopulmonary rehabilitation) [38].

With a focus on South Africa, YLDs rates in the country were extracted for all the rehabilitation-sensitive conditions considered. These YLDs rates were then ranked to identify the 11 conditions contributing the most to YLDs in South Africa, shown in Table 1. These 11 conditions contributed almost half (45%) of the total YLDs counts in 2017 (above six million). Some conditions were aggregated before the ranking exercise: for example, all upper and lower limb fractures and dislocations were combined into a single category, cardiovascular disease and heart failure were combined, and stroke was synthesized separately from cardiovascular disease. Stroke was considered separately given its relevance to local circumstances – just prior to the COVID-19 pandemic, stroke care was proposed as a national priority by the South African National Department of Health (non-communicable disease unit), due to the large increase in strokes over recent years (e.g. in association with HIV) and given that it is one of the leading causes of disability in South Africa [40].

**Table 1:** Top 11 conditions according to rank

Rank	Condition
1	HIV/AIDS (including resultant TB)
2	Chronic respiratory disease
3	Diabetes mellitus type 2
4	Age-related and other hearing loss
5	Musculoskeletal disorders (including low back pain)
6	Neonatal disorders
7	Congenital birth defects
8	All lower limb (LL), upper limb (UL), spinal and multiple fractures and dislocations
9	Cardiovascular diseases (excluding stroke) and heart failure
10	Stroke
11	Burns

#### Extraction of GBD estimates

We subsequently (in March 2020) extracted the age-standardized rates, and total YLDs count for the above conditions, for 28 years (1990–2017) to describe trends over time. We also extracted the 2017 all ages prevalence (number of people in the population with the condition), to provide an epidemiological indicator for the predominance of the condition in the South African population regardless of severity or sequelae. All selected estimates were imported from the GBD Webtool [31] to Microsoft Excel spreadsheets for storage, management, analysis and graphics generation. We summed YLDs of all fractures and dislocations. As in a previous analysis [18], we separated stroke as an independent reporting line from CVD estimates and included heart failure with CVD estimates. We calculated the relative proportionate contribution of the selected 11 conditions to the collective count of the conditions in 1990, 2017 and 2022 (predicted).

We calculated and reported prevalence rate per 100,000 for 2017 and 2022 based on the total population of South Africa and GBD estimates [41].

#### Analysis

The syntheses of GBD estimates were graphically depicted using frequency plots. The Mann-Kendall trend test was used for detecting monotonic trends of health condition profile. The analysis was performed in R Statistical Software [42] using the Kendall package, which has a function named *Mann-Kendall* for implementing the non-parametric test for monotonic trend detection [43]. The *p*-value associated with the Mann-Kendall test was used to test for the presence or absence of a statistically significant upward or downward trend in the condition profile. To compute a 95% bootstrap confidence interval (CI) for the slope of the trend, the *boot.ci()* function in the boot package was used [44]. Kendall’s tau statistic was used for testing the hypotheses “H_0_: no trend in health condition profile” versus “H_A_: monotonic trend in health condition profile (upward or downward)”. The non-parametric Mann-Kendall test has been used for trend analyses in health sciences [45] and was deemed appropriate for the current analysis since the GBD estimates do not have “seasonal trends” (fulfilling a requirement for Mann-Kendall analysis) and one point for a specific GBD metric is available per year.

We then calculated the regression line of best fit for each condition, specifically using estimates from 2012 to 2017. A five-year period for forecasting was most feasible since it enabled the future trend depiction on most recently available estimates. Underpinning the forecast on a longer retrospective period could have compromised predicted value validity, considering the transitioning healthcare system in South Africa and major transformations, such as the spin-offs after the introduction of free highly active antiretroviral therapy (HAART) [46]. In addition, the current rapid changes in global health profiles due to the COVID-19 pandemic do not underscore the relevance of longer predictions. As a means to forecasting, we extrapolated the estimates to 2022 using the best-fit rates of change from 2012 to 2017 and imposing the relevant best-fit formulae line onto the 2017 estimates (to predict 2018), then on the 2018 estimates (to predict 2019) and so forth. The predictions assumed that the rate and direction will be unchanged. The detailed method for the extrapolations is described in Additional File 1.

## Results

### YLDs counts per condition

**Fig. 1:**
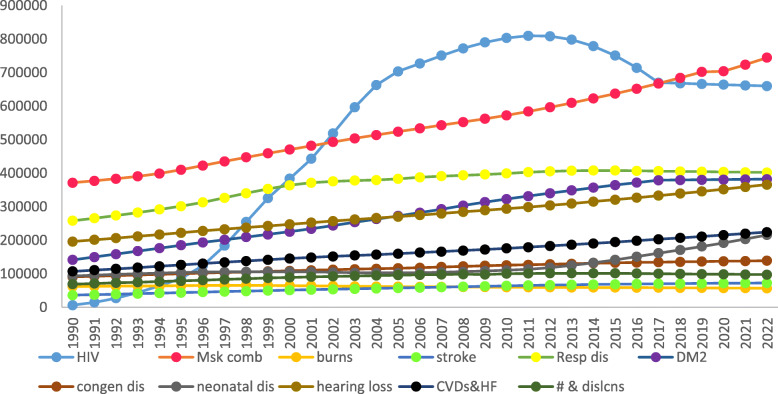
Trends of YLDs counts per condition year (1990–2017) and extrapolated as predicted values (2018–2022). Legend: # & dislcns = fractures and dislocations; congen dis = congenital birth defects; CVD & heart failure (HF) (excluding stroke); HIV (including resultant TB); Msk comb = Musculoskeletal disorders; neonatal dis = neonatal disorders; resp. dis = chronic respiratory disease

Figure 1 presents total YLDs trends (i.e., YLDs counts) in South Africa per condition year (1990–2017) and extrapolated as predicted values from 2018 to 2022. The actual values underpinning the trend graph calculation for YLDs are presented in Additional File 2.

**Fig. 2:**
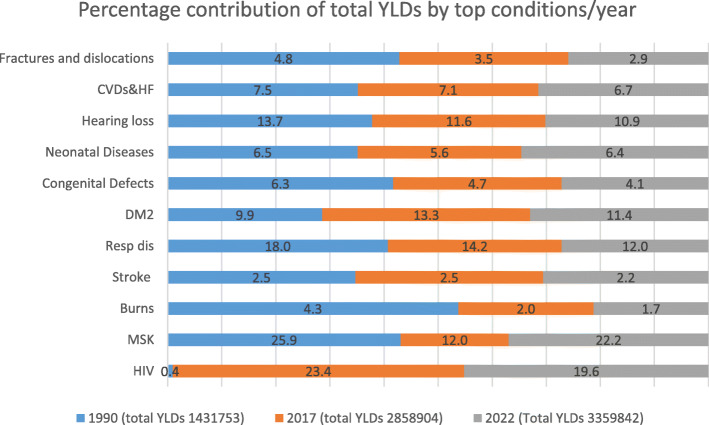
Relative proportionate contribution of the selected 11 rehabilitation amenable conditions (percentage of YLDs) to collective YLDs counts in 1990, 2017 and 2022 (predicted). Legend: CVDs& HF (excluding stroke); MSK = Musculoskeletal disorders; resp. dis = chronic respiratory disease

Figure 2 presents the proportionate contribution of the 11 top-ranked conditions to the total YLDs counts by these top conditions in 1990, 2017 and 2022 (predicted). Total YLDs count of the 11 selected conditions increased notably from above 1.4 million YLDs in 1990 to above 2.8 million YLDs in 2017 (an increase of approximately 100%). We predicted that the total YLDs count could amount to above 3.3 million in 2022 (reflecting a 17% increase over 5 years since 2017).

For HIV, the relative burden increased substantially after 1990 from 0.4% of the total YLDs to 19% in 2017. A slight reduction of about 1% in YLDs linked to HIV is predicted over the next 5 years.

### Age-standardized rates of YLDs

**Fig. 3:**
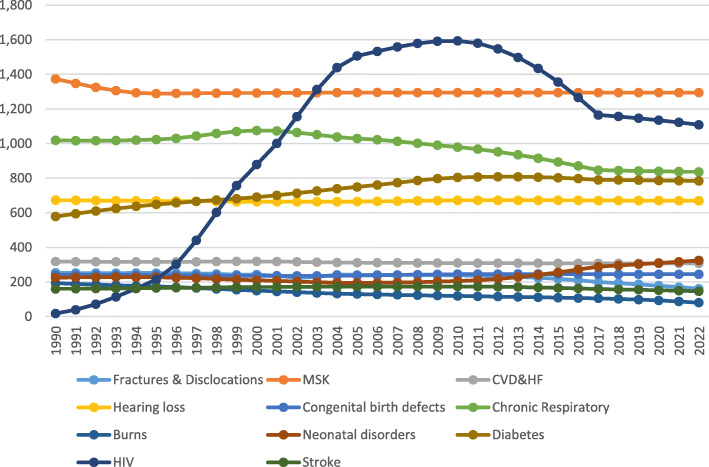
Age-standardized rates of YLDs per 100,000 years by rehabilitation amenable condition. Legend: Chronic respiratory = chronic respiratory disease; CVDs& HF (excluding stroke); MSK = Musculoskeletal disorders

Trends in the age-standardized YLDs rates are plotted in Fig. 3. Visually, the trends in the rates of all conditions (except HIV and perhaps chronic respiratory disease as well as DM2) appear relatively stable.

A comparison of age-standardized YLDs rates per condition is presented in Additional File 3, showing that the conditions that contribute the most to YLDs are biased towards adults (except for neonatal disorders).

The significance of trend changes in age-standardized YLDs rates per condition is reported in Table 2 as Mann-Kendall trend test estimates (tau), the associated 95% CIs and *p*-values. The Mann-Kendall trend tests suggested that there were significant trend changes over the past 28 years in YLDs rates for nine conditions and that the trends of four conditions (burns, chronic respiratory, cardiovascular disease, and fractures) have reduced over time.

**Table 2:** Mann-Kendall trend test estimates of age-standardized YLDs rates per rehabilitation amenable condition

Condition	Estimate (tau)	P-value	95% Confidence Interval
Stroke	0.407	0.003	−0.463, 0.569
HIV/AIDS (including resultant TB)	0.667	< 0.0001	−0.467, 0.577
Diabetes mellitus type 2	0.873	< 0.0001	−.437, 0.589
Neonatal disorders	**−0.02**	**0.855**	−0.538, 0.527
Burns	−0.900	< 0.0001	−0.625, 0.396
Chronic respiratory disease	−0.492	< 0.0001	−0.596, 0.514
Congenital birth defects	0.339	0.01	−0.591, 0.511
Hearing loss	**0.212**	**0.100**	−0.507, 0.559
Cardiovascular disease & heart failure (exclude stroke)	−0.76	< 0.0001	−0.568, 0.390
Musculoskeletal disorders	**0.132**	**0.300**	−0.393, 0.531
Fractures and dislocations	−0.661	< 0.0001	−0.540, 0.341

Legend: Trend changes were not significant for neonatal and musculoskeletal disorders, shown in bold

### Prevalence

Conditions had different projected directions of change and changes in prevalence from 2017 to 2022 (Table 3). Relatively larger increases in prevalence rate are predicted for neonatal, cardiovascular diseases and hearing loss. Relative larger decreases in the prevalence of fractures and dislocations as well as strokes are expected up to 2022.

**Table 3:** Predicted prevalence of common rehabilitation amenable conditions in 2022 based on South Africa’s population in 2017 [47]

Condition	Number of people per 100,000 in 2017	Number of people per 100,000 in 2022 (predicted population of 61.5 million [48])	% change (95% CIs)
HIV/AIDS (including resultant TB)	12,726	12,516	−1,7 (− 1.9 to − 1.4)
Burns	2764	2792	1.0 (0.7 to 1.5)
Stroke	889	855	−3.7 (− 5.2 to − 2.6)
Musculoskeletal disorders	6742	7004	3.9 (3.5 to 4.4)
Chronic respiratory disease	6126	6175	0.8 (0.6 to 1.1)
Diabetes mellitus type 2	7653	7750	1.3 (1.1 to 1.6)
Congenital birth defects	1761	1734	−1.0 (−1.7 to −0.6)
Neonatal disorders	1802	2089	15.9 (14.2 to 17.7)
Hearing loss	18,271	19,013	4.1 (3.8 to 4.4)
Cardiovascular disease & heart failure (excluding stroke)	5866	6177	5.3 (4.7 to 5.9)
Fractures and dislocations	3406	3167	−7.0 (−7.9 to −6.2)

## Discussion

To our knowledge, this is the first paper that reports on a synthesis of GBD estimates for rehabilitation-sensitive conditions that contribute most to YLDs in South Africa. The use of different GBD estimates (prevalence and YLDs) shows that different stories can be told for conditions, which range from communication and sensory conditions, to internal medical and musculoskeletal conditions. The most appropriate information on conditions requiring rehabilitation must thus be factored into the design and implementation of the South African NHI to ensure that the need for rehabilitation is recognized and funding is directed to where it is most needed. The South African-specific GBD estimates highlight the variability of rehabilitation needs in South Africa’s top-ranked conditions, and the potential burden they impose on individuals and society. They underscore the importance of undertaking in-depth, country-specific population-rehabilitation analysis to understand current need and potentially inform resource planning and investment.

Total YLDs per condition per year increased almost two-fold from 1990 to 2017, as the YLDs counts increased in all conditions except HIV (gradual decrease of HIV YLDs from 2004). The increase in YLDs counts may be explained by the dynamic interplay of local factors over time. One of these is fluctuations in South Africa’s life expectancy, which decreased by an average of 10 years between 1993 and 2003. Roll-out of antiretroviral treatment for HIV in 2004 [49] reversed the downward spiral of life-expectancy, which returned to 63 in 2017 [50]. The increase in life-expectancy in association with annual population growth may thus have contributed to the total number of YLDs increasing in South Africa, reflecting that many more people are now living for longer with infectious diseases.

The proportionate contribution of each condition to total YLDs count was dramatically transformed by HIV in 2017, compared to 1990. HIV currently affects one in five adults in South Africa [51]. Thus, in South African clinical and policy contexts, considering HIV separately from other common conditions requiring rehabilitation may be artificial, as HIV is often the underlying pathology for other conditions. These include musculoskeletal pain and trauma (such as amputations due to vascular impairments), stroke, DM2, neonatal disorders and hearing loss [9, 52–54]. While it appears as if the current proportionate condition contribution to total YLDs will remain stable, biological and clinical knowledge about potential interactive effects between co-existing conditions is important for health system design. Our synthesis is descriptive only, provided per singular condition. Although GBD estimates are corrected for comorbidity, the methodology assumes independence between individual diseases [32]. It therefore does not account for shared risk factors or diseases that may put one at risk of developing another disease [55]. More refined methods are under development and will most likely be used in future [56]. Moreover, the current GBD estimates do not reflect the legacy of the unprecedented COVID-19 global pandemic, which sees many people with unexpected rehabilitation needs [57–59]. Therefore considering compounded effects of disease patterns and new emerging conditions on functioning are paramount in the design, type, and adaptability of South Africa’s rehabilitation services.

Changes in the age-standardized YLDs rates varied between conditions over the 28-year study period. The rates of burns, chronic respiratory disease, cardiovascular disease and fractures appeared to have decreased over time (Fig. 3) and may reflect improved access to healthcare by more South Africans since the first democratic election in 1994. Decreasing trauma rates (fractures and burns) may also reflect improved housing and the impact of public health prevention campaigns. Whilst decreasing trauma rates to 2017 did not necessarily reflect improved access to rehabilitation, it is important to maintain this momentum of change, as trauma rates in South Africa remain alarmingly high and place an excessive burden on an already-constrained healthcare system [4]. Universal access to rehabilitation through the NHI should increase opportunities for injured South Africans to optimize function, regain dignity, and achieve community integration.

The predicted prevalence for most conditions (see Table 3) appears to be relatively stable until 2022, which supports informed service design. The discrepancy in terms of decreasing YLDs trend and slightly increased predicted prevalence noted for CVD may be due to advances in management and consequently lower disability levels; despite more people being diagnosed and/or perhaps inadequate risk factor control [60]. Despite the relative stability observed for most conditions, more information about functional impairment, condition severity and duration, functional impairments/disability type, ability to detect people who need rehabilitation, barriers to access and health-seeking behavior is necessary for rehabilitation service design. GBD uses DWs to incorporate disability severity and duration [33]. However, the validity of GBD DWs in lower-resourced settings such as South Africa has been questioned [61]. DWs are derived from research, self-report data and expert panels, which may not reflect the reality of rehabilitation for many people living in poorer socio-economic circumstances within a specific context (country). Thus, while GBD provides an opportunity to dissect prevalence and disease burden on a global macro level, it is essential that national and individual micro needs are also understood when planning and costing rehabilitation care packages. Economists and legislators must understand the continuum of rehabilitation need for priority chronic conditions and be able to estimate the greatest, most impactful population need on the continuum. It is also important that they consider the potential for people who receive rehabilitation to improve, and how ‘optimum function’ for these people can be described and measured.

### Rehabilitation workforce and educational implications

The projected prevalence and YLDs reported in this paper indicate that rehabilitation needs in South Africa will be substantial by 2022. To ensure that rehabilitation is an essential component of the NHI, there needs to be an adequately-sized, trained and recognized rehabilitation workforce that is sufficiently funded to address the needs of the poor and uneducated, who are mostly cared for in the public sector. South Africa’s capacity to mobilize such a workforce is probably inadequate. Tertiary-trained health professionals have traditionally delivered rehabilitation across the healthcare continuum. They take five or more years to train, and once graduated, they require high salaries. The number practising in the public sector has always been far fewer than the private sector, despite the significantly-greater need in the public sector [8, 13, 26]. The NHI budget is unlikely to be elastic, thus it is important that in aspiring to Rehabilitation 2030’s goals, South African policy makers and health funders remain realistic in how rehabilitation is best introduced into the NHI, how it is rationed, and how its impact is measured. Reliance on trained healthcare providers to deliver rehabilitation is, by default, going to limit access for many people. Delivering rehabilitation equitably in the NHI will require innovation – including task-shifting, educating, empowering and using patients, families, communities, and skilled and semi-skilled community health workers, to nurture the rehabilitation philosophy and functional restoration so they can work alongside healthcare professionals to optimize patient function [30].

Moreover, there are significant training implications for healthcare professionals at undergraduate and postgraduate levels to ensure consistent uptake of, and advocacy for, rehabilitation messages. Whilst undergraduate programs teach students about clinical assessment and treatment techniques, there is less focus on the skills required for rehabilitation. To achieve the rehabilitation millennium goals, health professionals providing rehabilitation must upskill to repackage their care to encompass health education, community health literacy facilitation, patient advocacy, and patient and community empowerment [62]. Moreover, they will need to use different ways of disseminating information to deliver their messages, such as social media, internet advice; and internet/telephone counselling.

### Limitations

This study was a synthesis of freely available GBD estimates. GBD condition-specific DWs may be constrained by geographical, socio-economic, and contextual issues; and do not necessarily reflect the context-specific reality of rehabilitation for many people in lower-resourced settings such as South Africa. Neither YLDs nor prevalence enable understanding of the core functional limitations that are experienced by South Africans. At the time when we conducted the study, the 2019 GBD estimates were not yet available, but our projections align well with these 2019 estimates. Thus, our main findings on trends in rehabilitation need in South Africa are not compromised. The GBD statistical modelling and estimates are dependent on good quality and sufficient local input data, which is very limited in lower income settings such as South Africa. We also did not report on sex-specific estimates, as preliminary analyses did not show any sex bias for any of the conditions included in this paper. This could be explored again in further research. Finally, as an initial description of South Africa’s rehabilitation needs, this study was not designed to distinguish between adults and children, as the top conditions contributing to YLDs in South Africa is biased to adults (except for neonatal disorders; see age distributions in Additional File 3) and do not provide an accurate reflection of the rehabilitation needs of children. Such differentiation was not the current objective, but is worth examining in future analyses to guide current and future service strategies.

## Conclusion

This synthesis of trends in GBD estimates indicate that rehabilitation needs in South Africa are potentially massive and unmet. This highlights a need to move towards innovative, adaptable, and context-specific rehabilitation, considering not only current local needs but also the projected changes in needs. The results of this paper, including limitations, should be considered when making decisions about funding for rehabilitation within the NHI and other aid funds, for rehabilitation workforce investments and developments.

## Supplementary Information

**Additional file 1.** YLDs total.

**Additional file 2.** Methods of extrapolation.

**Additional file 3.** 1990, 2017 and 2019 comparison: GBD age-standardized YLDs rates.

## Data Availability

The Global Burden of Disease (GBD) Study estimates supporting the conclusions of this article is available in the Institute for Health Metrics and Evaluation (IHME) GBD Results Tool | Global Health Data Exchange, http://ghdx.healthdata.org/gbd-results-tool

## Abbreviations

COVID-19: Coronavirus disease
TB: Tuberculosis
WHO: World Health Organization
NHI: National Health Insurance
BRICS: Brazil, Russian Federation, India, China, and South Africa
GBD: Global Burden of Disease
BoD: Burden of Disease
YLDs: years lived with disability
UL: upper limb
LL: lower limb
CI: confidence interval
CVD: Cardiovascular disease
DM2: type 2 diabetes
HR: human resources
DW: disability weight/ weighting
