# Supplementary material for: Towards a needs-based design of the physical rehabilitation workforce in South Africa: trend analysis [1990–2017] and a 5-year forecasting for the most impactful health conditions based on global burden of disease estimates
Source: BMC Public Health. 2021 May 13;21:913. doi: 10.1186/s12889-021-10962-y (PMC8116643; doi:10.1186/s12889-021-10962-y)
Supplement: Supplementary file 1 — Additional file 1. YLDs total. [file 12889_2021_10962_MOESM1_ESM.docx]

**Towards a needs-based design of the physical rehabilitation workforce in South Africa: trend analysis [1990-2017] and a 5-year forecasting for the most impactful health conditions based on Global Burden of Disease estimates**

**Louw Q^1*^, Grimmer K^1^, Berner K^1^, Conradie T^1^, Bedada DT^2^ and Jesus TS^3^**

**AUTHOR DETAILS**

^1^Division of Physiotherapy, Department of Health and Rehabilitation Sciences, Faculty of Medicine and Health Sciences, Stellenbosch University, Cape Town, South Africa

^2^Division of Epidemiology and Biostatistics, Department of Global Health, Faculty of Medicine and Health Sciences, Stellenbosch University, Cape Town, South Africa

^3^Global Health and Tropical Medicine (GHTM) & WHO Collaborating Centre for Health Workforce Policy and Planning, Institute of Hygiene and Tropical Medicine - NOVA University of Lisbon (IHMT-UNL), Rua da Junqueira 100, Lisbon 1349-008, Portugal

**Additional File 1**

**Methods of extrapolation**

1. The overall shape of the known estimates from 1990–2017 was described graphically.
2. The % change in estimates each year since 2012 was calculated to indicate recent change over time.
3. The % change was graphed for 2012–2017 and the type of line of best fit for this subset of estimates was identified. The appropriate equation for the line of best fit was determined.
4. The slope element of the relevant equation was then applied to the known % change from 2017 to estimate % change for each subsequent year (2018–2022).
5. The estimated rate for 2018–2022 was then calculated by adjusting the rate from the preceding year by the extrapolated incremental rate change between the preceding year and the next. For instance, the known rate in 2017 was adjusted by the extrapolated incremental rate change between 2017–2018 to estimate the 2018 rate. Then the extrapolated rate in 2018 was further adjusted by the extrapolated incremental rate change between 2018–2019 to estimate the 2019 rate, and so on.

| **Condition** | **Line of best fit for yearly % change 2012-2017** | **Equation used for calculation (did not include the intercept)** |
| --- | --- | --- |
| Stroke (CVA) | Linear, decreasing | Y=-0.25x |
| HIV | Linear, decreasing | Y= -1.34x |
| Type 2 Diabetes | Linear, decreasing | Y = -0.21x |
| Neonatal disorders | Logarithmic | Y = 1.38*(log x) |
| Burns | Linear, decreasing | Y = -0.98x |
| Chronic respiratory diseases | Linear, decreasing | Y= -0.26x |
| Congenital birth defects | Linear, decreasing | Y = -0.08x |
| Hearing loss | Linear, decreasing | Y = -0.03x |
| Cardiovascular disease & heart failure | Linear, increasing | Y = 0.008x |
| Musculoskeletal conditions | Linear, decreasing | Y = -0.0004x |
| Fractures & dislocations | Linear, decreasing | Y=-0.798x |
