# Supplementary material for: Towards a needs-based design of the physical rehabilitation workforce in South Africa: trend analysis [1990–2017] and a 5-year forecasting for the most impactful health conditions based on global burden of disease estimates
Source: BMC Public Health. 2021 May 13;21:913. doi: 10.1186/s12889-021-10962-y (PMC8116643; doi:10.1186/s12889-021-10962-y)
Supplement: Supplementary file 2 — Additional file 2. Methods of extrapolation. [file 12889_2021_10962_MOESM2_ESM.docx]

**Towards a needs-based design of the physical rehabilitation workforce in South Africa: trend analysis [1990-2017] and a 5-year forecasting for the most impactful health conditions based on Global Burden of Disease estimates**

**Louw Q^1*^, Grimmer K^1^, Berner K^1^, Conradie T^1^, Bedada DT^2^ and Jesus TS^3^**

**AUTHOR DETAILS**

^1^Division of Physiotherapy, Department of Health and Rehabilitation Sciences, Faculty of Medicine and Health Sciences, Stellenbosch University, Cape Town, South Africa

^2^Division of Epidemiology and Biostatistics, Department of Global Health, Faculty of Medicine and Health Sciences, Stellenbosch University, Cape Town, South Africa

^3^Global Health and Tropical Medicine (GHTM) & WHO Collaborating Centre for Health Workforce Policy and Planning, Institute of Hygiene and Tropical Medicine - NOVA University of Lisbon (IHMT-UNL), Rua da Junqueira 100, Lisbon 1349-008, Portugal

| **ADDITIONAL FILE 2**  **THE VALUES UNDERPINNING THE TREND GRAPH CALCULATION FOR YLDS** |
| --- |
| **YLDs N** |

|  | **HIV** | **Msk comb** | **burns** | **stroke** | **Resp dis** | **DM2** | **congen dis** | **neonatal dis** | **hearing loss** | **CVDs&HF** | **# & dislcns** |
| --- | --- | --- | --- | --- | --- | --- | --- | --- | --- | --- | --- |
| 1990 | 6346.548 | 371437.2 | 62217.3 | 36107.6 | 258255 | 141268.42 | 90544.5 | 93219.616 | 196172.8 | 107127.6 | 69056.5 |
| 1991 | 14564.58 | 377156.6 | 62535.1 | 37507.7 | 265698.7 | 149911.05 | 92490.4 | 95784.092 | 201214.6 | 110748.4 | 70882.9 |
| 1992 | 27289.2 | 383206.8 | 62930.9 | 38969.1 | 273663.5 | 158713.01 | 94414.9 | 98106.704 | 206315.7 | 114498.9 | 72919.2 |
| 1993 | 43939.58 | 390398.8 | 63336.8 | 40466.4 | 282508.7 | 167606.68 | 96327.4 | 100169.05 | 211565.8 | 118375.4 | 74684.6 |
| 1994 | 63923.78 | 399066.1 | 63801 | 42016.7 | 291981.7 | 176394.66 | 98240.1 | 101931.15 | 216930.8 | 122349.5 | 76819.8 |
| 1995 | 86588.57 | 410159.6 | 64408.8 | 43581.2 | 301617.6 | 185004.72 | 100170.2 | 103507.74 | 222390.8 | 126388.3 | 78802.1 |
| 1996 | 124335.3 | 422807.2 | 64957.3 | 45180 | 313248.4 | 193451.04 | 102062.1 | 104847.3 | 227829.9 | 130469.5 | 81019.0 |
| 1997 | 183909.1 | 435187.2 | 65190 | 46725.3 | 326423 | 201454.23 | 103765.0 | 105540.89 | 232878.9 | 134324.4 | 83176.5 |
| 1998 | 254845.8 | 447311.6 | 65149 | 48219.6 | 340152.9 | 209336.58 | 105330.6 | 105734.71 | 237751.1 | 138097.7 | 85283.0 |
| 1999 | 325671.8 | 459160.3 | 64881.3 | 49706.1 | 353151.8 | 217254.82 | 106939.6 | 105836.71 | 242682.9 | 141867.4 | 87278.2 |
| 2000 | 383638.7 | 470441.6 | 64546.1 | 51112.6 | 363903.4 | 225307.96 | 108624.1 | 105938.07 | 247593.3 | 145414.5 | 89114.4 |
| 2001 | 443033.6 | 481825.2 | 64067.1 | 52479.5 | 371409.5 | 234223.17 | 110399.2 | 105468.63 | 252589.2 | 148656.4 | 90730.8 |
| 2002 | 518655.8 | 493011.4 | 63286.1 | 53769.6 | 375556.9 | 243847.81 | 112073.7 | 104286.54 | 257333.0 | 151572.1 | 92215.3 |
| 2003 | 596772.6 | 503749.3 | 62421.6 | 55042.4 | 377948.1 | 253694.78 | 113559.9 | 103076.43 | 261819.8 | 154382.7 | 93499.6 |
| 2004 | 663002.9 | 513927.6 | 61697.7 | 56242.8 | 379681.2 | 263320.62 | 115006.2 | 102283.88 | 266159.3 | 157111.7 | 94648.4 |
| 2005 | 703640.3 | 523670 | 61424.9 | 57426.4 | 383053.7 | 272430.14 | 116425.4 | 102259.21 | 270416.1 | 159994.6 | 95779.7 |
| 2006 | 727159.8 | 533474.6 | 61333.9 | 58764.5 | 387830.1 | 282059.66 | 118068.2 | 103113.1 | 274950.2 | 163174.9 | 96870.2 |
| 2007 | 750850.6 | 543032 | 61069.9 | 60057.4 | 391059.4 | 292584.07 | 120062.0 | 104518.28 | 279691.3 | 166214.4 | 97804.2 |
| 2008 | 772326.3 | 552606.4 | 60688.7 | 61365.2 | 393713.1 | 303354.48 | 122136.7 | 106295.63 | 284554.2 | 169326.3 | 98653.3 |
| 2009 | 790145 | 562224.6 | 60261.3 | 62630.9 | 396217.4 | 313670.85 | 124022.0 | 108232.92 | 289329.5 | 172477.4 | 97804.2 |
| 2010 | 803178 | 572489.8 | 59959.4 | 63868.6 | 399257.1 | 322796.21 | 125581.6 | 110396.14 | 294013.5 | 175707.9 | 100070.9 |
| 2011 | 809840.6 | 584043.9 | 59749 | 65108.8 | 402859 | 331501.97 | 126878.1 | 113447.74 | 298896.2 | 179344.1 | 100789.1 |
| 2012 | 808444.6 | 596509.2 | 59555.8 | 66211 | 405615.6 | 340130.7 | 128285.0 | 118224.88 | 304044.6 | 182957.3 | 101350.6 |
| 2013 | 798221.8 | 609614.1 | 59353.9 | 67208 | 407309.8 | 348682.43 | 129786.2 | 124784.91 | 309501.6 | 186701.9 | 101550.4 |
| 2014 | 778806.5 | 623080.4 | 59123 | 68110.2 | 408161.1 | 356790.05 | 131302.7 | 132723.54 | 315259.5 | 190546.8 | 101589.7 |
| 2015 | 750713.9 | 637049 | 58858.8 | 68820.2 | 407861.4 | 364481.88 | 132533.3 | 141512.68 | 320921.0 | 194339.0 | 101376.7 |
| 2016 | 714340.6 | 651748.3 | 58569.5 | 69530 | 407120 | 371845.72 | 133663.0 | 151075.22 | 326841.2 | 198530.8 | 100931.9 |
| 2017 | 669977.4 | 667457.2 | 58298.3 | 70135 | 406078.7 | 378909.85 | 134637.9 | 161307.3 | 332933.5 | 202927.1 | 100320.7 |
| 2018 | 667959.4 | 684143.7 | 58024.3 | 70696 | 405266.5 | 379667.67 | 135580.4 | 170985.74 | 339259.3 | 206985.6 | 99718.8 |
| 2019 | 665940.4 | 701931.4 | 57751.6 | 71190.9 | 404456 | 380275.14 | 136393.8 | 181244.88 | 345705.2 | 211125.3 | 99120.5 |
| 2020 | 663920.4 | 703826.6 | 57474.4 | 71689.2 | 403647.1 | 380883.58 | 137212.2 | 192119.57 | 352273.6 | 215347.9 | 98525.7 |
| 2021 | 661899.4 | 723533.7 | 57198.5 | 72119.4 | 402839.8 | 381454.9 | 138035.5 | 203646.75 | 358966.8 | 219654.8 | 97934.6 |
| 2022 | 659877.4 | 744516.2 | 56924 | 72552.1 | 402034.1 | 382027.08 | 138863.7 | 215865.55 | 365787.2 | 224047.9 | 97347.0 |
