# Supplementary material for: Towards a needs-based design of the physical rehabilitation workforce in South Africa: trend analysis [1990–2017] and a 5-year forecasting for the most impactful health conditions based on global burden of disease estimates
Source: BMC Public Health. 2021 May 13;21:913. doi: 10.1186/s12889-021-10962-y (PMC8116643; doi:10.1186/s12889-021-10962-y)
Supplement: Supplementary file 3 — Additional file 3. 1990, 2017 and 2019 comparison: GBD age-standardized YLDs rates. [file 12889_2021_10962_MOESM3_ESM.docx]

**Towards a needs-based design of the physical rehabilitation workforce in South Africa: trend analysis [1990-2017] and a 5-year forecasting for the most impactful health conditions based on Global Burden of Disease estimates**

**Louw Q^1*^, Grimmer K^1^, Berner K^1^, Conradie T^1^, Bedada DT^2^ and Jesus TS^3^**

**AUTHOR DETAILS**

^1^Division of Physiotherapy, Department of Health and Rehabilitation Sciences, Faculty of Medicine and Health Sciences, Stellenbosch University, Cape Town, South Africa

^2^Division of Epidemiology and Biostatistics, Department of Global Health, Faculty of Medicine and Health Sciences, Stellenbosch University, Cape Town, South Africa

^3^Global Health and Tropical Medicine (GHTM) & WHO Collaborating Centre for Health Workforce Policy and Planning, Institute of Hygiene and Tropical Medicine - NOVA University of Lisbon (IHMT-UNL), Rua da Junqueira 100, Lisbon 1349-008, Portugal

**Additional file 3:** 1990,2017 and 2019 comparison: GBD age-standardized YLDs rates
